# The Role of Anthocyanins in Cardiovascular Health: A Review

**DOI:** 10.2174/011573403X372621250716123921

**Published:** 2025-07-21

**Authors:** Sangeeta Yadav, Alka Sharma, Sonam Bishnoi, Mukesh Gaur, Devyani Tomar, Akash Kumar

**Affiliations:** 1 Department of Food Technology, Guru Jambheshwar University of Science and Technology, Hisar, India;; 2 MMICT & BM (Hotel Management), Maharishi Markandeshwar (Deemed to be University), Mullana, India

**Keywords:** Nitric oxide, blood pressure, quality of life, risk factors, lipoproteins, bio-pigment

## Abstract

Anthocyanins are natural polyphenols found in various fruits and vegetables, offering numerous health benefits. Clinical studies suggest that anthocyanin supplementation may regulate blood pressure, improve lipid profiles, reduce triglycerides (TG), thiobarbituric acid reactive substances (TBARS), cytokines, and platelet aggregation, while also reducing arterial stiffness. The multiple pathways, including the downregulation of proinflammatory markers and suppression of the nuclear factor kappa-light-chain-enhancer of activated B cells (NF-κB) pathway, prevention of lipoprotein oxidation, enhancement of nitric oxide (NO) bioavailability, improvement of endothelial function, and modulation of the gut microbiota, collectively contribute to managing cardiac health. However, some clinical studies have found no significant positive impact of anthocyanins on cardiovascular disease, possibly due to the varied form, stability, dosage, and study duration. Therefore, future research should investigate anthocyanin stability, establish standardised therapeutic strategies, and conduct large-scale longitudinal studies to elucidate the impact of anthocyanin consumption on cardiovascular health and quality of life.

## 1 INTRODUCTION

Cardiovascular diseases (CVDs) are the leading cause of mortality worldwide, and these diseases include a group of disorders related to the heart and blood vessels. These include coronary heart disease, congenital heart disease, rheumatic heart disease, deep vein thrombosis, peripheral arterial disease, cerebrovascular disease, and pulmonary embolism [1]. The risk factors for CVDs include tobacco and alcohol addiction [2-4], unhealthy diet, inadequate physical activity [5], high blood pressure, high blood cholesterol, and high blood sugar or glucose [6]. The prevention and treatment of CVDs include medications, heart valve surgery, pacemakers, lifestyle changes, coronary angioplasty, coronary artery bypass graft surgery, radiofrequency ablation, thrombectomy, and blood transfusion [7]. By 2050, the prevalence of clinically diagnosed CVD is projected to reach 45 million adults in the United States, and 184 million adults (>61%) are estimated to have CVD along with hypertension [5]. Similarly, the projected incidence of CVD has increased drastically. This leads to an increase in economic burden [1].

Therefore, there is a need to prevent the risk of CVDs. In this context, diet modification plays a crucial role. A study revealed that low fruit consumption is increasing globally, increasing the population-attributable fraction of CVD mortalities and disability-adjusted life years [8]. Similarly, a systematic review and meta-analysis of prospective cohort studies have revealed that consuming fruits and vegetables is associated with a low risk of CVDs [9]. This may be due to bioactive compounds such as polyphenols, specifically anthocyanins. They have the potential to protect against CVD by contributing to cardiovascular health benefits [10] and exerting cardioprotective effects [11] by inhibiting the inflammatory process [12], improving endothelial dysfunction [13], and regulating nitric oxide (NO) production [14]. Anthocyanins exert antioxidant [14-16], anti-inflammatory [15], and antithrombotic effects [17]. Numerous studies have highlighted the association between anthocyanin consumption and a lower risk of cardiovascular diseases (CVDs) [13, 18-25].

A systematic review and meta-analysis of 32 randomised controlled trials investigated the effects of anthocyanins on cardiometabolic health. The interventions included purified anthocyanins, composite anthocyanin extracts, and antho-cyanin-rich foods. The dosage of anthocyanins ranged from 2.2 mg to 742 mg per day, and the intervention durations varied between 2 and 24 weeks. They found that anthocyanin consumption resulted in a significant reduction of fasting glucose levels (effect size: -0.31; 95% CI: -0.59, -0.04), postprandial glucose levels (effect size: -0.82; 95% CI: -1.49, -0.15), HbA1c (effect size: -0.65; 95% CI: -1.00, -0.29), total cholesterol (TC) (effect size: -0.33; 95% CI:-0.62, -0.03), LDL (effect size: -0.35; 95% CI: -0.66, -0.05). The HDL-C was increased, with an effect size of 0.24 (95% CI: -0.00, 0.49). However, fasting insulin levels, the homeostatic model assessment of insulin resistance (HOMA-IR), SBP, and diastolic blood pressure (DBP), and TG did not exhibit statistically significant changes. They also observed that anthocyanin supplementation did not lead to significant changes in inflammatory markers, including C-reactive protein, TNF-α, and IL-6 [26].

A meta-analysis analysed 44 randomised clinical trials with 2,353 subjects and 15 prospective cohort studies with 5,54,638 subjects, focusing on the evaluation of the effects of anthocyanins and anthocyanin-rich berries on cardiometabolic health. The study found that purified anthocyanins significantly reduced LDL-C by 5.43 mg/dL (95% CI: -8.96, -1.90 mg/dL; *p =* 0.003), TG by 6.18 mg/dL (95% CI: -11.67, -0.69 mg/dL; *p =* 0.027), and TNF-α by 1.62 pg/mL (95% CI: -2.76, -0.48 pg/mL; *p =* 0.005) while increasing HDL-C by 11.49 mg/dL (95% CI: 7.43, 15.55 mg/dL; *p <* 0.001). The blueberry intake increases HDL-C by 1.46 mg/dL (95% CI: 0.20, 2.72 mg/dL; *p =* 0.023) and cranberry consumption reduces BMI by 0.30 kg/m^2^ (95% CI: -0.57, -0.02 kg/m^2^; *p =* 0.035). It was observed that anthocyanin consumption had no significant effect on systolic blood pressure (SBP) or diastolic blood pressure (DBP). However, anthocyanin intake was associated with a lower incidence (17%) of coronary heart disease and a reduced risk of total CVD by 27%. Additionally, higher anthocyanin consumption was associated with a lower risk of total CVD mortality (9%) [18].

A meta-analysis examined 47 randomised controlled trials investigating the effects of anthocyanin supplementation or consumption on cardiometabolic risk factors. The study found that anthocyanin supplementation significantly reduced BMI by -0.21 kg/m^2^ overall. However, the effect of anthocyanins may vary depending on the delivery method, such as supplements, which decreased BMI by 0.35 kg/m^2^, while food increased BMI slightly by 0.1 kg/m^2^. However, no statistically significant effect was observed on waist circumference. The anthocyanin supplement/extract reduced fasting blood glucose (FBG), with the effect being more pronounced in type 2 diabetes patients (-15.33 mg/dL) than in non-diabetic participants (-1.55 mg/dL). However, no significant effect on fasting serum insulin was observed. Across all subgroups, there was a significant reduction in TG of -9.46 mg/dL. The average decrease in TC and LDL was -6.98 mg/dL and -6.91 mg/dL, respectively. In individuals with dyslipidemia, TC and LDL-C levels were reduced by -13.41 mg/dL and -10.68 mg/dL, respectively. The dietary supplementation with anthocyanins resulted in a significant increase (+3.94 mg/dL) in apolipoprotein A; however, no significant effect was observed on apolipoprotein B. The meta-analysis suggests that anthocyanins, specifically in supplement form, can improve cardiometabolic risk factors [27].

Jang *et al.* conducted a systematic meta-analysis to explore the effect of anthocyanins on blood lipids. The study involved 2,788 participants and found that anthocyanin supplementation significantly reduced triglyceride (TG) levels (SMD = -0.10; 95% CI: -0.18 to -0.01; I^2^ = 34%, *p =* 0.01) and low-density lipoprotein cholesterol (LDL-C) levels (SMD = -0.16; 95% CI: -0.26 to -0.07; I^2^ = 38%, *p =* 0.01), and increased high-density lipoprotein-cholesterol (LDL-C) levels (SMD = 0.42; 95% CI: 0.20 to 0.65; I^2^ = 81%, *P <* 0.01). While TC levels exhibited a non-significant reduction (SMD = -0.05; 95% CI: -0.12 to 0.01; I^2^ = 28%, *p =* 0.04) [28]. However, these reviews do not examine the mechanism by which anthocyanin provides these benefits.

In this context, Reis *et al*. and Mohammadi *et al*. conducted a well-detailed systematic review based on animal and human interventions. They demonstrated that anthocyanin consumption reduces oxidative stress markers, inhibits lipid peroxidation, and enhances total plasma antioxidant capacity. Additionally, anthocyanin-rich foods and extracts have been shown to lower LDL-C and TG, as well as cardiovascular biomarkers associated with endothelial dysfunction, while increasing HDL-C levels. These articles also discussed the mechanisms through which anthocyanin offers these benefits [21, 29].

While well-detailed systematic reviews on the effects of anthocyanins on cardiovascular health, oxidative stress, and inflammation have been published, there is still a need for comprehensive narrative reviews. Systematic reviews adhere to strict inclusion and exclusion criteria, often focusing on specific study designs, types of interventions, or outcome measures. This approach may result in the omission of valuable findings from observational studies, mechanistic research, or emerging clinical trials that do not meet the predefined criteria. Thus, a narrative review should provide a broader range of evidence, mechanistic insights, and future directions which was left by systematic analyses.

This study aims to address this gap by examining the potential molecular pathways involved and summarising the clinical outcomes of recent clinical studies. This study comprehensively analyses established mechanisms, including antioxidant and anti-inflammatory properties, modulation of gut microbiota, and lipid profile. By elucidating the underlying mechanisms involved, this review will strengthen the foundation for future research and potentially lead to more effective dietary and therapeutic strategies for preventing CVD. This article also discussed the potential adverse effects of high consumption of anthocyanins. Additionally, the review highlights knowledge gaps, such as the need for well-designed randomised clinical trials, dose-dependent analyses, and long-term safety assessments.

## 2 METHODOLOGY

For this narrative review, a comprehensive literature search was conducted using various databases, including Web of Science, Scopus, Google Scholar, EBSCO, and PubMed. The keywords that were used for literature search were Anthocyanins, Anthocyanidin, Cyanidin, Delphinidin, Malvidin, Pelargonidin, Peonidin, Cardiovascular Diseases, Heart Disease, Atherosclerosis, Hypertension, Coronary Artery Disease, Stroke, Endothelial Dysfunction, Oxidative Stress, and Inflammation. The Boolean operators were used to combine terms for a more targeted search. The references of all included articles were also screened to identify relevant studies. Those articles that are peer-reviewed, published in the English language, and focus on the effects of anthocyanins on CVDs were included in this study. Irrelevant articles were excluded from the study. From the selected studies, data were extracted into the various sections: type of study, subject, number of subjects, dosage/duration, and outcome. The extracted data were discussed narratively in the section “Clinical Evidence for the Cardiovascular Benefits of Anthocyanins” and represented in the table. Additionally, to support the aim of this narrative review, various sections, including “Anthocyanins: Chemistry, Sources, and Metabolism, Mechanisms of Anthocyanin Action in Cardiovascular Health, Adverse Effects of Anthocyanins, and Limitations and Future Research Directions”, were also discussed. However, the major limitation of this review is the absence of a formal quantitative synthesis (meta-analysis), the restriction to English-language publications, and the publication bias.

### 2.1 Anthocyanins: Chemistry, Sources and Metabolism

Anthocyanins are water-soluble pigments found in the flowers, seeds, fruits, and leaves of numerous plants [30]. A total of 702 different anthocyanins and 27 anthocyanidins are present in nature. Still, only six anthocyanidins are available for the human body: cyanidin, delphinidin, pelargonidin, peonidin, malvidin, and petunidin [31], as shown in Fig. (**1**). Anthocyanins are glycosides composed of an anthocyanidin aglycone plus one or more glycosidically bonded mono- or oligosaccharidic units. Their basic skeleton comprises a flavylium cation backbone with two benzene rings connected by three carbon atoms to form a C6-C3-C6 skeleton [32]. Even if these molecules contain an oxonium group in their structure, the flavonoid skeleton maintains its ring nomenclature with the charged oxygen atom on the C ring and is susceptible to nucleophilic attack by the addition of compounds such as SO_2_, ascorbic acid, H_2_O_2,_ or water [33]. The stability of anthocyanins depends on the B-ring in their structure and on the presence of hydroxyl or methoxyl groups [31]. The stability of pigments is influenced by pH, light, temperature, structure, metal ions, and oxygen. An acidic pH yields red pigments, whereas at a basic pH, it is converted into a blue pigment, making it unstable and degrading it into dark brown, oxidised compounds [33].

### 2.2 Sources of Anthocyanins

Anthocyanins are pigments responsible for the red, blue, and purple colors of many fruits and vegetables [34]. They are abundant in purple corn, blueberries, raspberries, bilberries, chokeberries, elderberries, red cabbage, red grapes, and pomegranates [35]. Owing to their high anthocyanin content (10 to 772.4 mg/100 g FW), berries are considered the primary source of anthocyanin, accounting for 39% and 43% of the total anthocyanin intake in the United States of America (USA) and Europe, respectively [36]. Individuals who follow the Mediterranean diet consume more anthocyanins than those who do not [37]. The average daily intake of anthocyanins is estimated to be 12.5, 19.8, 24.2, and 64.9 mg per person in the United States [38], Netherlands, Australia, and Italy, respectively [36]. The recommended daily intake of anthocyanins is not established; however, 49-133 mg/day is well tolerated by an average healthy adult [39].

### 2.3 Metabolism of Anthocyanins in the Human Gut

Glycosides of anthocyanidins are present in the form of anthocyanins. After ingestion, anthocyanin absorption occurs at different gastrointestinal tract sites, including the oral cavity, stomach, small intestine, and colon [40], where different metabolic activities can lead to anthocyanin degradation and metabolism [41]. Fig. (**2**) represents the metabolism of anthocyanins. The initial digestion of anthocyanins begins in the oral cavity, where saliva degrades 8 to 90% of the anthocyanin, which entirely depends on the structure of the anthocyanin [41, 42]. The oral cavity contains a microbiota that produces digestive enzymes, such as β-glucosidase, to convert the glycosidic group into aglycones and transform the anthocyanin structure into its corresponding chalcone [35, 36]. Oral surface epithelial cells and salivary gland terminal ducts secrete efflux transport enzymes, second-stage enzymes, and hydrolases, which degrade anthocyanins into different products [35]. Intestinal enzymes for phase 2 metabolism and enteric recycling of anthocyanins are present in the oral cavity [35] and enter the alimentary canal with anthocyanins for further digestion and absorption [41]. The oral digestion of anthocyanins is limited because anthocyanins remain in the oral cavity for a short time.

The undigested anthocyanins in the oral cavity enter the stomach through the upper alimentary canal. The stomach's acidic (pH 1.5-4) gastric juice provides favorable conditions for absorbing anthocyanins, allowing them to persist in glycoside form. This absorption might depend on uridine diphosphate glucuronosyltransferase and sulfamic acid transferase in gastric tissues [41]. Due to the cell membrane's passive diffusion, anthocyanin is transported through different routes by an organic anion membrane carrier named bilitranslocase, which is present in the gastric mucosa, vascular endothelium, kidneys, and liver [36]. After metabolising, anthocyanins enter the circulatory system through the liver and are transferred to the intestine with bile [41]. This transporter rapidly absorbs anthocyanins in plasma within 30 minutes of ingestion through both portal and general circulation, thereby inhibiting the transport of quinoidal forms [40].

Studies have shown that glucose transporter 1 and glucose transporter 3 are effective glucose transporters involved in the gastric absorption of anthocyanins [35]. Only 1%~10% of anthocyanins are absorbed by gastric epithelial cells in a complete structure due to their short stay in the stomach, and approximately 10~20% of anthocyanins are actively transported in the form of primary metabolites through gastric epithelial cells. Numerous studies have shown that the rate of anthocyanin absorption in the stomach can reach 20%, even though it is an essential part of anthocyanin absorption [41].

The small intestine is the primary site of anthocyanin absorption in the intestinal tract, occurring *via* both passive transport and active transport by intestinal epithelial cells [35]. It undergoes deglycosylation (*i.e*., cleavage of the glycoside) by β-glucosidase and lactase-phlorizin hydrolase in the intestinal lumen, increasing its hydrophobicity and thereby facilitating its entry into epithelial cells by passive diffusion [36]. Alternatively, sodium-dependent glucose transporter 1 (SGLT1) or glucose transporter 2 (GLUT2) may facilitate the active transport of intact glycosides into epithelial cells; however, the involvement of SGLT1 in anthocyanin absorption remains uncertain [35]. The absorption of the acylated form is four times lower than that of the nonacylated form. Further metabolic detoxification of glycosylated anthocyanin occurs for many xenobiotics, which increases their hydrophilicity and facilitates elimination from the body through bile and urine. In the liver, anthocyanins are metabolised by phase I (oxidation, reduction, and hydrolysis reactions), and phase II metabolites, such as glucuronic, methylated, and sulfate metabolites, which are known as phase 2 metabolites catalysed by enzymes (catechol-O-methyltransferase, sulfotransferase and uridine-5’-diphosphoglucuronosyltransferase), are responsible for conjugation reactions in the intestines, liver, and kidneys [42, 43]. Consequently, after consuming anthocyanin-rich food, sulfated, methylated, and glucuronidated anthocyanins are found in human plasma and urine [35].

Alternatively, before conjugation, anthocyanin aglycones are degraded to phenolic acids and aldehydes within the intestinal lumen or epithelial cells. The undigested anthocyanins enter the colon and provide the same pH as the small intestine. The microbial flora present in the colon, including Eubacterium, Bacteroides, and Bifidobacterium, produce deglycosylation enzymes that cleave sugars, resulting in aglycones that further undergo ring opening to produce various phenolic acids or aldehydes [41, 35]. Methylated phenolic acids, such as vanillic acid (VA) or ferulic acid, can increase plasma concentrations (1-2 mM) within 15 hours of anthocyanin consumption and can be detected in plasma after 48 hours of ingestion [42]. The metabolised phenolic acids have numerous health benefits, including antioxidant, anti-inflammatory, and antitumor effects [41]. Consequently, the concentration of phenolic acids increases with decreasing ingested anthocyanin concentration in the gastrointestinal tract. The products of degraded anthocyanins are transported through epithelial monocarboxylic acid transporters to the liver or kidneys. In contrast, products metabolised through the intestinal flora can be absorbed by epithelial cells and enter the blood circulation. Unabsorbed anthocyanins are excreted in the feces, or they can be reabsorbed, primarily in the colonic mucosa. The colon metabolises anthocyanins by cleaving glycosidic bonds and breaking down the anthocyanin heterocycle, resulting in the production of phloroglucinol derivatives and benzoic acid. Other byproducts of anthocyanin breakdown in the large intestine include catechol, pyrogallol, resorcinol, tyrosol, 3-(3’-hydroxyphenyl) propionic acid, dihydrocaffeic acid, and 3-(4’-hydroxyphenyl) lactic acid. These metabolic products have a regulatory effect on the growth of probiotics in the intestine, influencing the development of intestinal microbiota [44].

### 2.4 Mechanisms of Anthocyanin Action in Cardiovascular Health

The effective functioning of the cardiovascular system is closely linked to the functioning of the endothelium. Endothelium impairment is related to the development of cardiovascular risk factors such as hypertension, dyslipidemia, and atherosclerosis [45, 46]. An elevation in the endothelium's prothrombotic and proinflammatory properties, with reduced endothelial vasodilatation, characterises this condition [47].

Monocytes migrate into the subendothelial region, differentiating into activated macrophages and engulfing oxidised lipoproteins, forming foam cells. The gradual accumulation of these foam cells contributes to plaque deposition, leading to atherosclerotic lesions and CVD development [46]. Anthocyanins support cardiovascular health by enhancing antioxidant activity that effectively neutralises free radicals, reduces inflammatory stimuli, prevents the activation of inflammation-related signaling pathways, regulates the production of anti-inflammatory agents, and alleviates inflammatory responses. This may contribute to a reduction in endothelial dysfunction and atherosclerosis [44]. Numerous studies suggest that anthocyanins impact nitric oxide levels, regulate blood vessel contraction, protect cardiovascular walls, and decrease platelet aggregation, all of which contribute to a reduced risk of CVDs [48]. Fig. (**3**) illustrates the proposed mechanism by which anthocyanins mitigate CVDs.

#### 2.4.1 Anthocyanin and Antioxidative Activity

Oxidative stress is a major contributor to CVDs. Oxidative stress leads to neutrophil infiltration, increased protease secretion, and the production of oxidative intermediates, including reactive oxygen species (ROS). Nicotinamide adenine dinucleotide phosphate (NADPH) oxidase (NOX2 and NOX4) and xanthine oxidase-1 (XO-1) are responsible for generating ROS [49]. NOX2 plays a crucial role in the immune response by generating superoxide; however, it may contribute to oxidative stress when overexpressed. NOX4 expression in endothelial cells and fibroblasts produces hydrogen peroxide, which influences redox signalling and vascular health. XO1 catalyses the hypoxanthine and converts it into xanthine, followed by the production of uric acid. During this reaction, superoxide and hydrogen peroxide are produced as byproducts. The excessive production of ROS may lead to oxidative stress, endothelial dysfunction, and inflammation. Additionally, the enzymatic oxidation of phospholipids or LDLs results in oxidised products such as oxidised LDL (ox-LDL), which further causes molecular damage and acts as an indicator of endothelial dysfunction and atherosclerosis [21]. All these factors may contribute to the onset of CVDs.

In this context, anthocyanins have emerged as promising antioxidants with the potential to mitigate oxidative stress [40]. The antioxidant activity of anthocyanins relay upon its structure, and the properties are influenced by (i) the B ring catechol moiety; (ii) the number of -OH group; (iii) the hydroxylation and methylation pattern; (iv) acylation; (v) glycosylation; and (vi) the oxonium ion present in the C ring [50]. Due to the hydroxyl groups in the B-ring and the oxonium ion in the c-ring, anthocyanins undergo the following mechanisms: (a) single electron transfer (SET), (b) hydrogen atom transfer (HAT), and (c) chelation of transition metals to eliminate free radicals [21]. A free radical (R•) converts to a more stable form by accepting hydrogen from an antioxidant (AH) through HAT mechanism, whereas, the SET mechanism involves an antioxidant donating an electron to the free radical, leading to the reduction of the oxidised species intermediate by bonding with reactive oxygen species (ROS) such as singlet oxygen (^1^O_2_), superoxide (O_2_^-^), hydroxyl radical species (.OH), and hydrogen peroxide (H_2_O_2_) [50]. Additionally, anthocyanins may scavenge ROS, inhibit NOX activity, and modulate the expression of superoxide dismutase (SOD) and glutathione peroxidase (GPx). They also promote translocating nuclear factor erythroid 2-related factor 2 (Nrf2) into the nucleus. Furthermore, modifications such as methylation, hydroxylation, and glycosylation of the anthocyanin B-ring structure can boost antioxidant activity, with anthocyanins containing a single sugar molecule demonstrating superior antioxidant capacity. The acylation of anthocyanins with phenolic acids significantly enhances their antioxidant potency, whereas 5-glycosylation tends to reduce the efficacy of their antioxidant properties [44]. Anthocyanins exhibit antioxidant activity similar to vitamin C and surpass all other antioxidants in the body [48]. Anthocyanins effectively induce vasodilatory effects by increasing endothelial nitric oxide synthase (eNOS) activity, which produces NO, and downregulating the vasoconstrictor angiotensin-converting enzyme (ACE) level. They also reduce the activity of oxidising enzymes such as xanthine oxidase and NADPH oxidase and thus prevent the formation of oxLDL [51]. Anthocyanins have been proven to modulate the expression of genes encoding both anti- and pro-oxidant enzymes [40]. The consumption of anthocyanin extract demonstrates strong antioxidant properties by enhancing capillary permeability and strength. This effect also inhibits platelet formation and accelerates the production of nitric oxide (NO), resulting in vasodilation [50]. Thus, anthocyanins may reduce the risk of hypertension, atherosclerosis, and myocardial infarction by reducing oxidative damage and inflammation.

#### 2.4.2 Anthocyanin and Anti-inflammatory Effects

An imbalance between oxidation and antioxidant defenses in the endothelium may disrupt the properties of endothelial cells, leading to the hyperaggregation of platelets at lesions and the formation of thrombi on vascular walls. These changes trigger thrombosis and inflammatory responses through various inflammatory signaling pathways, such as the NF-κβ pathway [21, 52]. These responses are regulated by several factors, including enzymes, cytokines, vasoactive mediators, and lipid mediators [53]. NF-κβ induces the expression of proinflammatory genes encoding cytokines such as tumor necrosis factor-alpha (TNF-α), monocyte chemoattractant protein-1 (MCP-1), interleukin-6 (IL-6), and interleukin-1 beta (IL-1β), promoting inflammatory responses [21]. The NF-κβ pathway is sensitive to oxidative stress and controls the gene expression of mediators responsible for the inflammatory response [54]. All such changes induced by the NF-κβ pathway led to the expression of cytokines and inflammatory enzymes, including iNOS and COX-2 [55]. Inducible nitric oxide synthase (iNOS) is a key proinflammatory enzyme that produces excessive NO and is associated with the progression of various inflammatory diseases [56, 57].

Cyclooxygenase (COX-2), an enzyme belonging to the oxygenase class, is a key proinflammatory enzyme responsible for the synthesis of lipid mediators, such as prostaglandin E2 (PGE2) [56-59]. Up-regulation of COX-2 expression is associated with vascular inflammation, endothelial dysfunction, and atherosclerosis. PGE2 plays a significant role in inflammatory responses and is synthesised when COX-2 metabolises arachidonic acid [60]. PGE2 modulates platelet aggregation and vascular smooth muscle cell proliferation, thereby contributing to atherogenesis and increasing the risk of hypertension, thrombosis, and myocardial infarction.

Anthocyanins exhibit cardioprotective effects by modulating COX-2/PGE2 signaling and reducing inflammation. Anthocyanins inhibit COX-2 expression, thus reducing the synthesis of PGE2 and other pro-inflammatory prostanoids [61]. They suppress the expression of NF-κB regulation, maintaining endothelial integrity, preventing vascular remodeling, and reducing the risk of atherosclerotic plaque formation [62]. Additionally, they can also enhance the controlled release of NO [63], which is crucial for healthy endothelial function, and its unavailability leads to endothelial damage [64].

Anthocyanins effectively induce vasodilatory effects by increasing endothelial nitric oxide synthase (eNOS) activity to produce NO and downregulate the vasoconstrictor angiotensin-converting enzyme (ACE) level [51]. They inhibit the release of pro-inflammatory cytokines and histamines, reducing Toll-like receptor 4 (TLR4) expression, cyclooxygenase activity, and hindering the activation of nuclear factor kappa B (NF-κB) and mitogen-activated protein kinases (MAPKs) signaling pathways [44]. They block TNF-α-induced inflammation in human endothelium through the inhibition of monocyte chemoattractant protein-1. Ischemia-reperfusion injury generates free radicals, which promote white blood cell adhesion to microcapillary walls, thereby reducing blood flow and contributing to capillary damage. Anthocyanins can neutralise these free radicals and help prevent capillary damage [50].

Anthocyanin decreases MCP-1 expression, a chemokine that controls leukocyte migration and infiltration. It also suppresses the expression of inflammatory cytokine genes by inhibiting the IKK/NF-κB pathway. Simultaneously, anthocyanins enhance adiponectin secretion, which lowers TNF-α and IFN-γ levels, boosts anti-inflammatory IL-10 production, and shifts macrophages from a pro-inflammatory to an anti-inflammatory phenotype. Anthocyanin-rich extracts significantly suppress the expression of proinflammatory genes (including those encoding iNOS, COX-2, IL-1α, IL-1β, IL-6, and IL-17) [65, 66]. Furthermore, leptin produced by adipocytes influences the immune response by activating immune cells and promoting pro-inflammatory mediators [67].

Cardiac hypertrophy develops in response to inflammation, driven by elevated levels of IL-6, IL-1β, and TNF-α. This condition is associated with the activity of the CTRP3/AMPK pathway. Cyanidin-3-glucoside (C3G) administration restored the changes in CTRP3/AMPK activity. Additionally, CTRP3 levels and the p-AMPK/AMPK ratio were significantly higher after administration of C3G, which is dose-dependent [68]. Through these mechanisms, anthocyanins play a crucial role in cardiovascular health by reducing inflammation, improving endothelial function, and lowering the incidence of CVD-related complications such as hypertension and thrombosis.

#### 2.4.3 Anthocyanin and Gut Modulation

Numerous studies have demonstrated a correlation between anthocyanins, gut microbiota, and cardiac health through various pathways, as shown in Fig. (**4**). Anthocyanins are not fully absorbed in the upper gastrointestinal tract; therefore, they reach the colon. They act as a prebiotic substrate, interacting with the gut microbiome to modulate gut microbiota (GM), regulate lipid metabolism, reduce inflammation, and mitigate the impact of harmful metabolites. Anthocyanin increases the abundance of *Enterococcus, Bacteroidetes, Bifidobacteria, Lactobacillus, Akkermansia muciniphila, Eggerthella lenta,* and *Faecalibacterium prausnitzii.* Protocatechuic acid, produced through the microbial metabolism of anthocyanin, is responsible for reducing the growth of pathogenic bacteria, including *Firmicutes, Helicobacter pylori, Clostridium perfringens,* and *Escherichia coli* [69, 70]. The increased abundance of beneficial bacteria leads to an increased production of short-chain fatty acids, which enhances intestinal integrity, reduces inflammation, and modulates lipid metabolism. SCFAs activate AMP-activated protein kinase (AMPK), thus suppressing lipid synthesis and promoting fatty acid oxidation. FXR plays a crucial role in synthesising, transporting, and utilising lipids [71, 72]. Studies have demonstrated that anthocyanin consumption downregulates FXR expression, resulting in reduced weight gain, lipid accumulation, and adipose tissue deposition [72, 73]. Additionally, downregulation of FXR expression reduces lipid synthesis and accumulation. Anthocyanins consumption reduces the PPARγ expression and lipid accumulation in hepatic and adipose tissues [72].

High TMAO levels have been associated with an increase in systemic inflammation and risk of CVD. Anthocyanin supplementation reduces TMAO levels by decreasing the abundance of Enterobacteriaceae and influencing bile acid metabolism through the FXR signaling pathway. Additionally, they reduced the risk of atherosclerosis by reducing the TMAO levels. Anthocyanin downregulates the expression of inflammatory cytokines and cell adhesion molecules such as VCAM-1 and ICAM-1. Thus reduced the risk of plaque formation and the risk of atherosclerosis [72].

#### 2.4.4 Anthocyanins and Modulation of the Lipid Profile

Lipids, as lipoproteins, are distributed throughout the human body. These lipoproteins comprise phospholipids, proteins, TGs, and unesterified cholesterol [74]. The balance of these blood lipids is crucial for cardiovascular health. Lipid profiles, which typically include TC, LDL-C, HDL-C, and TG, provide valuable information about the risk of CVD. An imbalance in cholesterol fractions leads to a condition known as dyslipidemia. This condition is characterised by increased TC, TG, and LDL-C levels and reduced HDL-C. These elevated levels of lipoproteins contribute to the development of atherosclerotic plaques within the arterial wall and narrowing of the arterial lumen. This narrowing disrupts blood flow and significantly increases the risk of CVD [75, 76]. However, HDL-C exerts a positive and protective effect on cardiovascular health by enhancing reverse cholesterol transport (RCT). RCT is a process that modulates the removal of excess cholesterol and fat from the body by transporting them from the artery walls to the liver [21]. The mechanism of RCT involves the efflux of cholesterol, mediated by specific receptors, from peripheral tissues, including foam cells, macrophages, and atherosclerotic plaques. The receptors transport cholesterol to the liver, where it is eliminated as bile cholesterol or bile salts [77]. HDL-C is one of the principal cholesterol receptors in this pathway; therefore, its concentration determines the efficiency of RCT [75]. Therefore, increased levels of HDL-C reduce the risk of CVDs [21]. Moreover, the pathogenesis of atherosclerosis is mediated by the oxidative modification of low-density lipoprotein (ox-LDL) followed by the formation of macrophage-derived foam cells. Therefore, inhibiting oxLDL formation could be a potential target for effectively reducing the risk of atherosclerosis [78, 79].

Anthocyanins may inhibit several key enzymes involved in the synthesis and metabolism of fatty acids and triacylglycerols, including acyl-CoA synthase 1 (ACS1), fatty acid synthase (FAS), and glycerol-3-phosphate acyltransferase (GPAT). Moreover, they reduce the expression of sterol regulatory element binding protein (SREBP-1), which plays a role in lipase production and fatty acid synthesis. Secondly, anthocyanins stimulated the expression of genes involved in cholesterol metabolism, including cholesterol 7α-hydroxylase (CYP7A1) and 3-hydroxy-3-methylglutaryl-CoA reductase (HMG-CoAR). Third, anthocyanins may enhance adipocyte metabolism by regulating the transcription of adipose triglyceride lipase (ATGL) through the transcription factor FoxO1. Fourth, various studies have shown that anthocyanins reduce hepatic triglyceride accumulation and enhance lipid metabolism by activating AMPK signaling pathways, which are vital regulators of hepatic lipid metabolism. Fifth, anthocyanins also suppress the expression of adipogenic genes, such as PPARγ, while enhancing the expression of carnitine palmitoyltransferase (CPT-1) and PPARα, which are associated with fat oxidation [67].

Anthocyanins promote RCT by increasing the concentration of HDL-C in blood serum, which directly impacts the potential of these lipoproteins to facilitate overall cholesterol efflux [28]. Fruits and vegetables rich in anthocyanins effectively improve the lipid profile by eradicating excess cholesterol and reducing the risk of atherosclerosis by inhibiting oxLDL formation and improving endothelial dysfunction [80]. An investigation conducted by Jang *et al*. revealed a significant reduction in TC and TG levels after supplementation of the diet with anthocyanins [28]. Additionally, the anti-inflammatory effects of anthocyanins are protective against atherosclerosis [81], and the potential mechanism involves the inhibition of atherosclerotic progression [64].

### 2.5 Clinical Evidence for the Cardiovascular Benefits of Anthocyanins

Several studies have investigated the effects of various anthocyanin-rich fruits on cardiovascular biomarkers, as summarised in Table **1**. Okomota *et al*. conducted a double-blind study in which fourteen older adults (mean age 73.3 years) received either a placebo or two daily capsules of New Zealand blackcurrant (NZBC) extract (300 mg each, 35% blackcurrant content) for 7 days. The study revealed that NZBC administration significantly reduced carotid-femoral pulse wave velocity and central blood pressure (CBP), whereas no significant changes in serum lipids were detected [82]. The observed reduction in CBP without significant changes in serum lipid levels suggests that the intervention influenced vascular function rather than lipid metabolism. This effect may be attributed to improved endothelial function, increased NO bioavailability, reduced oxidative stress, and vascular inflammation.

A randomised, double-blinded trial by Whyte *et al*. investigated the effects of blueberry supplementation (500 or 1000 mg/day blueberry powder and 100 mg/day purified extract) on older adults aged 65-80 years. The study revealed that consuming a purified anthocyanin extract (100 mg/day) for 6 months significantly reduced systolic blood pressure (SBP) compared with the control group [83]. Bhaswant *et al*. investigated the impact of administering 250 ml of plum juice for 8 and 12 weeks, respectively. They reported a significant reduction in TNF-α and improved blood pressure, insulin sensitivity, and other metabolic risk factors. These results highlight the potential cardiovascular health benefits of diets rich in anthocyanins [84].

However, some studies have found no significant impact on cardiovascular biomarkers. For example, in a randomised controlled trial, blood orange juice (400 ml/day) was used as an intervention. They found that consuming juice for 2 weeks resulted in no significant changes in blood pressure, lipid profile, and hs-CRP [85]. Similarly, in another study, after 6 months of consuming 150 g of blueberries per day, HDL cholesterol levels, endothelial function, and arterial stiffness showed significant improvements. However, a lower dose (75 g/day) of blueberries had no significant benefits, and the study suggested a dose-dependent effect of anthocyanins. However, no significant changes were observed in insulin resistance, blood pressure, nitric oxide levels, or overall plasma thiol levels [86]. The effects of anthocyanins on HDL cholesterol levels, endothelial function, and arterial stiffness were dose-dependent. At higher dosages, anthocyanins may upregulate ATP-binding cassette transporters A1 (ABCA1) and G1 (ABCG1), thereby increasing HDL cholesterol levels. Additionally, higher intake of anthocyanins improves vascular relaxation and endothelial function. It also reduces vascular oxidative stress and downregulates pro-inflammatory cytokines, thus mitigating arterial stiffness. In contrast, the low dose cannot activate FXR signaling, reduce oxidative stress, and modulate lipid metabolism. In a study, Australian females and males with mild cognitive impairment consumed fruit juice (250 ml/day) with high and low anthocyanin dosage for 8 weeks. This may have resulted in a significant reduction of TNF-α, with no significant changes observed in microvascular function, IL-6, IL-1β, and C-reactive protein [87]. These findings underscore the need for further investigations with larger sample sizes and extended intervention periods to elucidate the long-term effects on CVD risk.

The studies discussed in Table **1** have demonstrated that anthocyanin supplementation has a complex impact on hypertension, with several studies finding reductions in systolic and diastolic blood pressure (DBP), while others report no significant effect. For example, blueberry and hibiscus extract interventions resulted in significant decreases in blood pressure, whereas anthocyanin-rich foods, such as blood orange juice and blackcurrant extract, improved vascular function parameters (flow-mediated dilation and pulse-wave velocity) without a significant impact on blood pressure. The heterogeneity in outcomes may be due to varied methodology, including study design quality (ranging from rigorous double-blinded to open-label protocols), intervention duration (4 days to 6 months), anthocyanin dosing (47-784 mg daily), delivery systems (capsules, powders, juices, whole fruits), and population characteristics (spanning diverse ethnicities, age groups, and baseline health statuses). Based on the discussed studies, clinical recommendations for dyslipidaemia management are ≥320 mg/day. Whereas supplementation of 300-320 mg daily is recommended for chronic inflammatory conditions. However, anthocyanins' potential to modulate blood pressure requires careful clinical consideration, particularly in individuals with hypertension. Thus, for the implementation of anthocyanins in a clinical setting, further elucidation is required through more rigorous, standardized, and longitudinal investigations.

### 2.6 Adverse Effects of Anthocyanins

Anthocyanin-rich foods and supplements are generally considered safe foods. However, few clinical studies have found adverse effects in populations consuming anthocyanins as an intervention. A study by Chan *et al*. found that consuming 1400 mg of anthocyanin supplementation leads to dark green stools in approximately half of the population. Additionally, poor sleep and mild headaches were also observed in some subjects; however, no serious adverse effects were observed [102]. The color of the stool may be changed due to the presence of metabolised products of anthocyanin. A study has demonstrated that anthocyanins may increase the monoamine neurotransmitters, including norepinephrine [104]. The increased level of norepinephrine may lead to mild stimulatory effects and cause poor sleep.

In another study, mild symptoms were found in two participants, including dizziness and insomnia, while five others reported dark stools [103]. Anthocyanins have a vasodilatory effect, and a high dosage may lead to a sudden drop in blood pressure, which can cause dizziness. Some participants were withdrawn from the clinical study due to adverse effects such as dark stool, insomnia, abdominal pain, diarrhea, dizziness, and skin rashes [105]. Several abnormalities were reported, including cold, headache, bruising, enteritis, and ulitis, from black soybean testa extracts [101]. Anthocyanins from blueberries and blackcurrants have been shown to cause adverse effects after consumption during the active period, including diarrhea, minor headaches of short duration, darker stools than usual, and nausea [106]. These symptoms were observed in a few participants and may be potentially influenced by other factors, including the production of IgE antibodies, the presence of additional compounds in anthocyanin-rich foods, and the high fiber content, which can cause digestive discomfort. Additionally, digestive issues resulting from sugar consumption, as well as potential interactions between anthocyanins and certain medications or other foods, may also contribute. Anthocyanin-rich foods, such as berries, contain various compounds that can trigger reactions in sensitive individuals, as well as reactions due to allergen cross-reactivity [107]. However, further research is required to elucidate the exact mechanisms underlying the adverse effects associated with anthocyanin consumption.

## 3 STUDY LIMITATIONS

Over the last few decades, anthocyanins have garnered significant interest and have been shown to exhibit promising cardioprotective effects. However, several limitations exist, such as their inherent instability, highlighting the need for further investigation to improve their stability for large-scale applications. The lack of standardised therapeutic strategies, such as effective dosage, bioavailability, and treatment duration, limits the utilisation of anthocyanins in clinical practices. Variability in study designs, population characteristics, and dietary sources of anthocyanins contributes to inconsistencies in outcomes.

Therefore, large-scale longitudinal studies are crucial for understanding how anthocyanins influence cardiovascular health and quality of life. Longitudinal studies have enabled researchers to identify the gradual impact of anthocyanin intake on heart health, including blood pressure, lipids, atherosclerosis, and overall well-being. *In vitro* studies with physiologically relevant designs must be used to investigate the effects of anthocyanins and their metabolites on gene, protein, and mRNA expression.

## 4 FUTURE RESEARCH DIRECTIONS

The mechanistic studies suggest that anthocyanins modulate oxidative stress, inflammation, and lipid metabolism. In contrast, the precise mechanistic pathways underlying this association are not well understood, specifically the relationship between anthocyanins and gut microbiota, their metabolites, and endothelial function. Furthermore, studies on personalised nutrition, synergistic interventions with other bioactive compounds, and long-term safety assessments are essential to translate findings into evidence-based dietary recommendations for preventing and managing CVD.

## CONCLUSION

This review suggests that anthocyanin-rich foods are a potential approach for promoting cardiovascular health. Clinical studies have suggested that anthocyanin supplementation may reduce CVD risk by regulating blood pressure, improving lipid profiles, attenuating oxidative stress, suppressing inflammatory biomarkers, ameliorating endothelial dysfunction, regulating vasodilator production, and improving gut microbiome. Anthocyanins exert their benefits through multiple mechanisms. These compounds may downregulate proinflammatory markers such as TNF-α, MCP-1, and IL-10, potentially by suppressing the NF-κB pathway. Additionally, anthocyanins have antioxidant properties, thus preventing lipoprotein oxidation. Anthocyanins may increase NO bioavailability and improve endothelial function, capillary permeability, and fragility. They also enhance the gut microbiota and intestinal integrity, reduce inflammation, and increase the production of protocatechuic acid and short-chain fatty acids.

## Figures and Tables

**Fig. (1):**
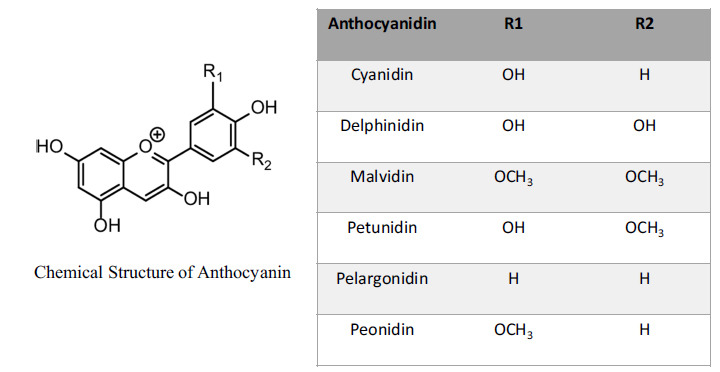
Basic structure of anthocyanins.

**Fig. (2):**
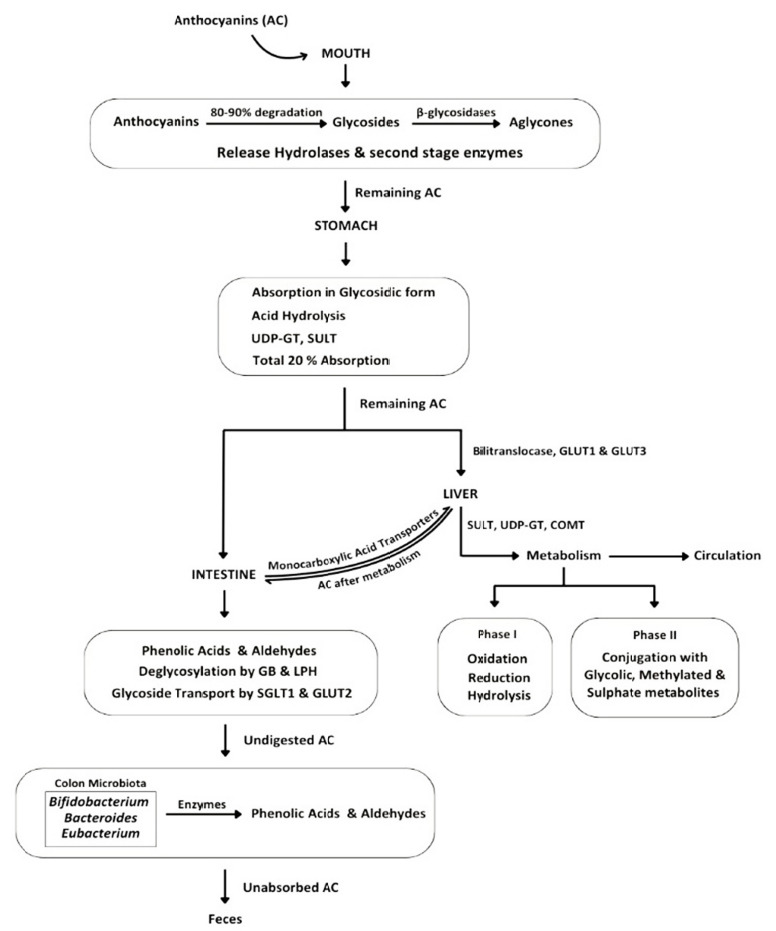
Metabolism of anthocyanins in the human body. **Abbreviations:** AC, Anthocyanin; UDP-GT, uridine-5’-diphosphoglucuronosyltransferase; SULT, sulfamic acid transferase; GLUT1, glucose transporter 1; GLUT3, glucose transporter 1; COMT, catechol-O-methyltransferase; GB, β-glucosidase; LPH, lactase-phlorizin hydrolase; SGLT1, sodium-dependent glucose transporter 1; GLUT2 glucose transporter 2.

**Fig. (3):**
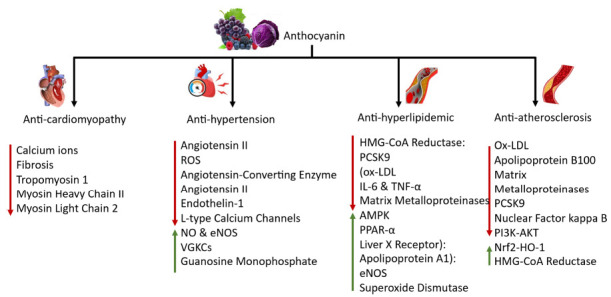
Proposed mechanism by which anthocyanins mitigate CVDs.

**Fig. (4):**
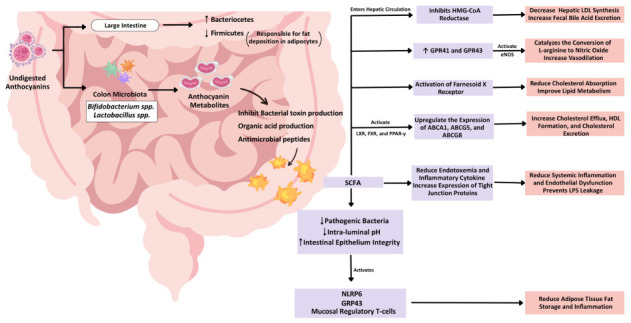
Potential mechanism of anthocyanin-driven gut microbiota modulation and SCFA-mediated cardiometabolic benefits. **Abbreviations:** SCFA, Short-Chain Fatty Acids; NLRP6, NOD-like receptor family pyrin domain containing 6; GRP43, G-protein-coupled receptor 43; HMG-CoA Reductase, 3-hydroxy-3-methylglutaryl coenzyme A reductase; GPR41, G-protein-coupled receptor 41; GPR43, G-protein-coupled receptor 43; ABCA1, ATP-binding cassette transporter A1; ABCG5, ATP-binding cassette subfamily G member 5; ABCG8, ATP-binding cassette subfamily G member 8.

**Table 1:** Clinical trials on anthocyanins and cardiovascular health.

**Type of Study**	**Subject**	**No. of** **Subjects**	**Dosage/Duration**	**Outcome**	**References**
Randomised, Double-blinded, Placebo-controlled study	Older Adults (65-80 years)	122	500-1000 mg Blueberry powder & 100 mg purified extract per day for 6 months	Intake of purified extract reduced SBP by -3.08 mmHg after 3 months and -6.99 mmHg after 6 months	[83]
Pre- and post-intervention of blood samples	Healthy Individuals (25 to 75 years)	26	320 mg/day Medox^®^ capsules for 28 days	Inhibition of platelet activity and aggregation Significant reduction in MPV, MCH, MCHC, and fibrinogen levels	[88]
Crossover, Randomised, Double-blind clinical trial	Older females and males (mean age 65.9)	16	250 ml/day of plum juice for 4 days	Improved postprandial vascular and microvascular function Reduced CRP levels and IL-6	[89]
Randomised controlled clinical trial, placebo-controlled group	Peri- and early postmenopausal women from Northeastern Connecticut (45-60 years)	38	392 mg/day (low anthocyanin food) & 784 mg/day (high anthocyanin food) for 6 months	Significantly reduced TG, IL-1β, and TBARS levels in a dose-dependent manner Improved dyslipidemia, reduced inflammation, and oxidative stress	[90]
Randomised, Controlled, Single-blind, Crossover trial	Healthy Caucasians men and Premenopausal women (20-45 years)	15	400 ml/day of blood orange juice for 2 weeks	Significantly increased flow-mediated dilation, urinary hesperetin-3'-glucuronide, and hesperetin-7-glucuronide No significant impact on blood pressure, lipid profile, and hs-CRP	[85]
Randomised controlled trials	Males and females (50-75 years)	115	75 g =1/2 cup 150 g = 1 cup blueberry for 6 months	1 cup/day of blueberries improved endothelial function and arterial stiffness Increased HDL No significant impact on insulin resistance, blood pressure, nitric oxide, or overall plasma thiol	[86]
Randomised, double-blind, placebo-controlled, and parallel trial	Females and males with diabetes Nephropathy (above 18 years) from Iran	60	425 mg *Hibiscus sabdariffa linnaeus* (HSL) for 8 weeks	Significantly reduced SBP, blood urea nitrogen, blood creatinine, urine creatinine, urine albumin levels, and high-sensitivity C-reactive protein (hs-CRP) levels. Significantly increased total antioxidant capacity levels No significant change in DBP, fasting blood glucose, and glomerular filtration rate.	[91]
Randomised double-blinded placebo-controlled trial	Patients with dyslipidemia from Guangzhou city	176	40,80 & 320 mg/day for 12 weeks	Dose-dependent reduction in plasma ceramides Significantly reduced plasma N-palmitoyl sphingosine (Cer 16:0) and N-tetracosanoylsphingosine (Cer 24:0) at 320 mg/day dose Reduced non-HDL-C, apolipoprotein B, and TC	[92]
Randomised, Double-blind, placebo-controlled, crossover study	Healthy Thai men (mean age 22 ± 3 years)	17	Two capsules of 300 mg blackcurrant extract for 7 days	Increased stroke volume and cardiac output, reduced systemic vascular resistance	[93]
Randomised, double-blind, placebo-controlled, crossover trial	Japanese older Adults (mean age 73.3 ± 1.7)	14	Two capsules of 300 mg containing 35% blackcurrant extract for 7 days	Reduced carotid-femoral pulse-wave velocity, central blood pressure, carotid-femoral pulse-wave velocity, and central blood pressure No significant changes in serum lipids.	[82]
Randomised, controlled, double-blind clinical trial	Australian females and males with mild cognitive impairment (mean age 75.3 ± 6.9 years)	31	250 ml fruit juice with high anthocyanin dose (201 mg) and low anthocyanin dose (47 mg) for 8 weeks	Reduction in Serum Tumor Necrosis Factor Alpha (TNF-α) No effect on Microvascular function Serum IL-6, IL-1β, and c-reactive protein	[87]
Open-label randomised, controlled clinical trial	Swedish Patients within 24 hours of percutaneous coronary intervention with standard medical therapy	50	40 g freeze-dried bilberry for 8 weeks	Significantly reduced TC, LDL & oxidised LDL (20%) compared with the controlled group. Significantly increased gallic acid, vanillic acid-4-O-sulfate, p-coumaric acid, caffeic acid 4-β-D-glucuronide, and caffeic acid 4-β-D-glucuronide in the plasma	[94]
Randomised crossover design clinical study	Healthy males and females	13	750 ml blackberry beverage for 14 days	Significant reduction in plasma level of TG (26.33%), TC (8.10%), and glucose level Slight increase in superoxide dismutase (SOD) and catalase (CAT) enzymatic activities	[95]
Randomised, Placebo-Controlled, Double blind, three arm Cross-Over Trial	Hyperlipidemic participants from Norwich, UK	52	Capsule containing 320 mg anthocyanin (bilberry trihydroxy-type or black rice dihydroxy-type)/day for 28 days	No effect on biomarkers of vascular function (total/HDL/LDL-C, triglycerides, and Apo B), glycemic control (glucose, fructosamine), HDL function (Apo A1, HDL3, PON1 aryl esterase, and lactonase activity), or plasma bile acids	[96]
Randomised, double-blind, crossover design	Healthy male trained cyclists (age 39 ± 10 years)	13	Two capsules a day containing 300 and 600 mg/day blackcurrant extract for 1 week	No effect on blood lactate levels, carbohydrate, and fat oxidation	[97]
Randomised, double-blind study	Australian Hypertensive overweight 15 males and 14 females (mean age 45 years)	29	250 ml/day of plum juice and raspberry cordial for 12 weeks	Reduced SBP (12±3 mm Hg), DBP (9± 2 mm Hg), insulin (6±3 pmol/L) and leptin (4±2.5 µg/ml) Improved adiponectin (3.62± 0.28 µg/ml) Changes in LDL, HDL, fasting plasma glucose, and C-peptide concentrations	[84]
Randomised, single-blind, parallel-group study	Madrid Men or Postmenopausal women between the ages of 45-85 years	59	5 g/day red berry mix for 12 weeks	Increased carbohydrate fermentation with reduced level of TC/HDL ratio, systolic, and DBP	[98]
Randomised three-arm controlled crossover design	Healthy men aged 50 to 70 years recruited at the University of California, Davis	12	240 ml/day red wine for 7 days	Reduced AI 13% (arterial stiffness), SBP (-4.1 mmHg), and DBP (-5.6 mmHg)	[99]
Randomised, blind, placebo-controlled study	Iraqi patients suffering from hypertension aged 50 to 65 years	28	Two capsules a day containing 300 mg anthocyanin extract for 30 days	No significant differences (*P*˂0.5) in mean blood pressures of patients, slight elevation in diastolic levels, significant lowering in total ROS concentrations. Significant decrease (*P*˂0.01) in C-reactive protein levels in the test group (3.1 mg/L)	[100]
Randomised, blind, placebo-controlled study	Iraqi patients suffering from diabetes mellitus aged 50 to 65 years	22	Two capsules a day containing 300 mg anthocyanin extract for 30 days	No significant reduction (*P*˂0.5) in blood glucose levels. Total ROS was reduced to 204.6 IU/mL Significant (*P*˂0.01) lowering of C-reactive protein levels to 5.3 mg/L	[100]
Randomised double-blind placebo-controlled clinical trial	Overweight Korean participants with body mass index (BMI >23) or waist circumference (WC >90 cm/males, >85 cm /females	63	Two capsules of black soybean testa extract thrice before meals with a total intake of 2.5 g/d	Significant decrease in lipid biomarkers, hematologic indicators for arteriosclerosis, and abdominal fat. Improve plasma lipid profiles	[101]
Randomised, double-blind, placebo-controlled, crossover study	Patients with T2D (HbA1c ≥7%) controlled with oral hypoglycemic agents in Hong Kong	20	Daily dose of four capsules, each containing 350 mg bilberry anthocyanin extract, for 4 weeks	Reduction in HbA1c values, whereas no significant changes in systolic and DBP, and fasting plasma glucose.	[102]
Randomised, double-blind, placebo-controlled trial	Chinese prediabetes or newly diagnosed, untreated type 2 diabetes patients aged 40-75 years	160	Two Medox capsules twice daily, containing 320 mg bilberry and blackcurrant anthocyanins for 12 weeks	Significant increases in serum adipsin (net change 0.15 µg/mL) and decreases in visfatin (-3.5 ng/mL). Improved HbA1c (-0.11%), apolipoprotein A-1 (apo A-1) (0.12 g/L) and apolipoprotein B (apo B) (-0.07 g/L) levels.	[103]

## LIST OF ABBREVIATIONS

Apo B: Apolipoprotein B
CAT: Catalase
COX-2: Cyclooxygenase-2
CVD: Cardiovascular Diseases
DBP: Diastolic Blood Pressure
GI: Gastrointestinal
HbA1c: Hemoglobin A1c
HDL-C: High-Density Lipoprotein Cholesterol
HSL: Hibiscus Sabdariffa Linnaeus
IL: Interleukin
LDL-C: Low-Density Lipoprotein Cholesterol
MCP-1: Monocyte Chemoattractant Protein-1
NF-κB: Nuclear Factor Kappa-light-chain-enhancer of Activated B Cells
NO: Nitric Oxide
PGE2: Prostaglandin E2
PON1: Paraoxonase-1
RCT: Reverse Cholesterol Transport
ROS: Reactive Oxygen Species
SBP: Systolic Blood Pressure
SOD: Superoxide Dismutase
TC: Total Cholesterol
TG: Triglycerides
TNF-α: Tumor Necrosis Factor-alpha
